# Supplementary material for: Determining population structure from k-mer frequencies
Source: PeerJ. 2025 Mar 5;13:e18939. doi: 10.7717/peerj.18939 (PMC11890038; doi:10.7717/peerj.18939)
Supplement: Supplemental Information 1 [file peerj-13-18939-s001.pdf]

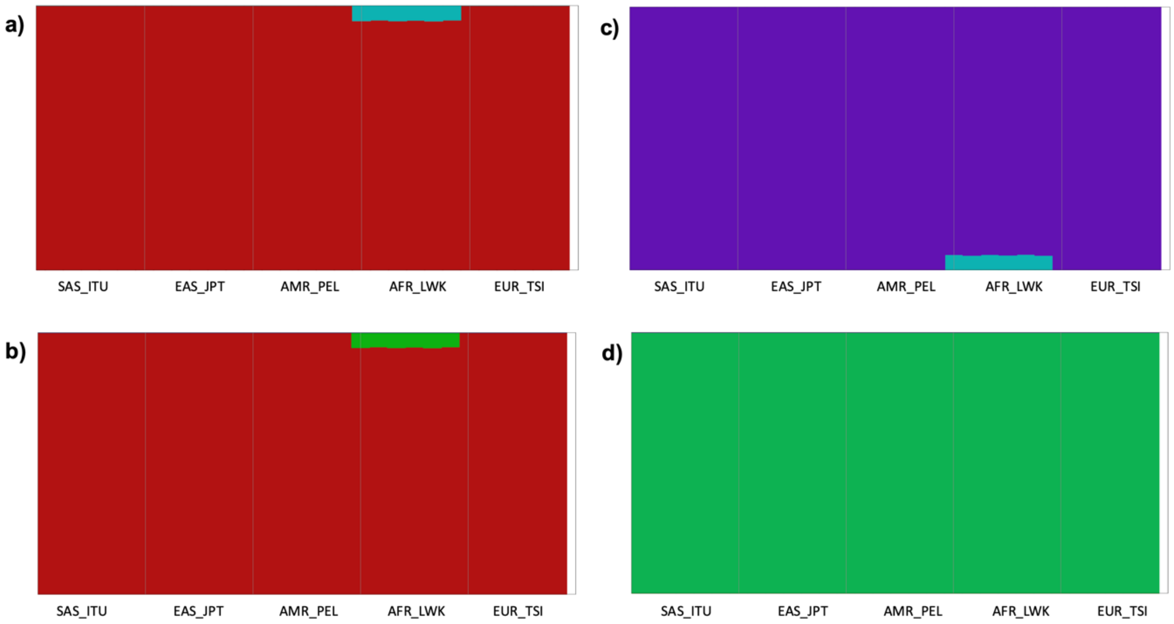

Figure A1: *fastStructure* assignment of individuals into a single population. *chooseK* method determined an optimal K between 1 and 5. a) fastStructure results for K = 2; b) fastStructure results for K = 3; c) fastStructure results for K = 4; d) fastStructure results for K = 5;

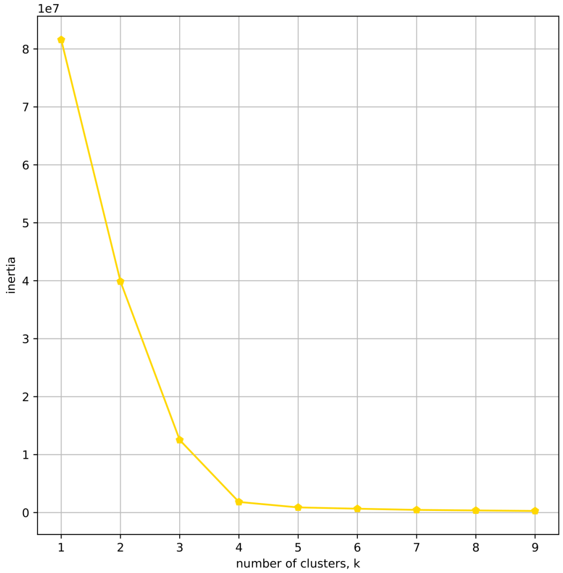

Figure A2: Scree plot showing a deterministic number of clusters = 4 ("elbow point") determined by K-Means using 2 PCs (13% of the variance) from k-mer frequencies of four superpopulations (excluding AFR).

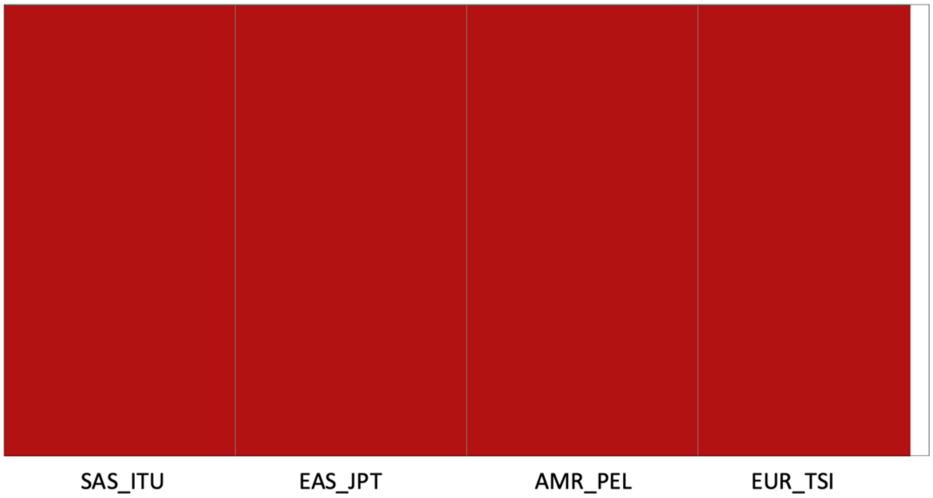

Figure A3: *fastStructure* assignment of individuals into a single population. *chooseK* method determined an optimal K between 1 and 2. fastStructure results for K = 2.

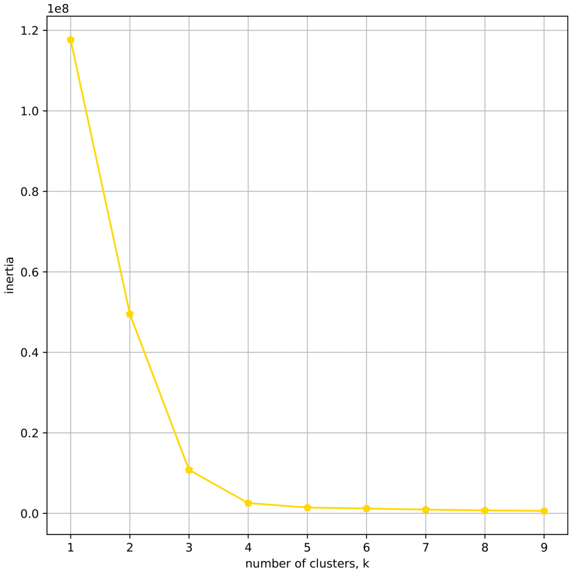

Figure A4: Scree plot showing a deterministic number of clusters = 4 (“elbow point”) determined by K-Means using 2 PCs (8.4% of the variance) from k-mer frequencies of four superpopulations with additional samples of multiple origin from EAS and EUR superpopulation.

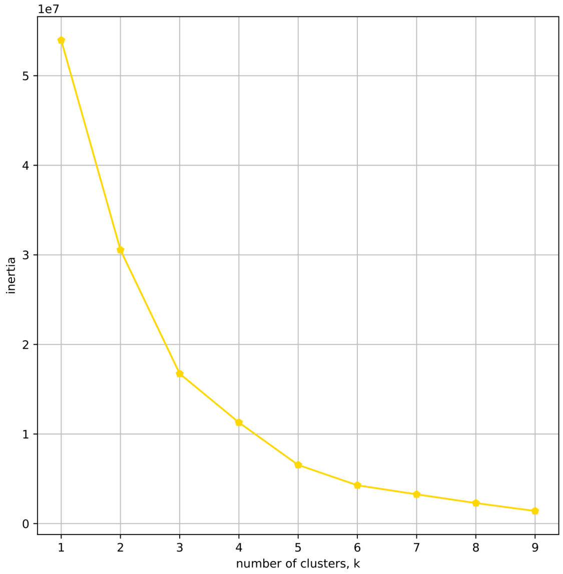

Figure A5: Scree plot suggesting three clusters using the "elbow point" determined by K-Means using 2 PCs (13% of the variance) from k-mer frequencies of three populations, CDX (single origin), CHB (multiple origin), and JPT (single origin). All three populations are of a single superpopulation (EAS).

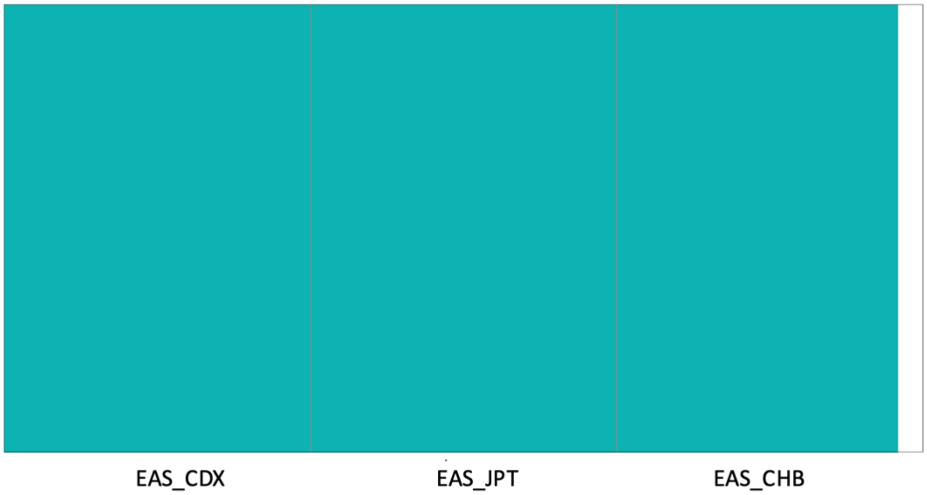

Figure A6: *fastStructure* assignment of individuals into a single population. *chooseK* method determined an optimal K between 1 and 2. fastStructure results for K = 2.

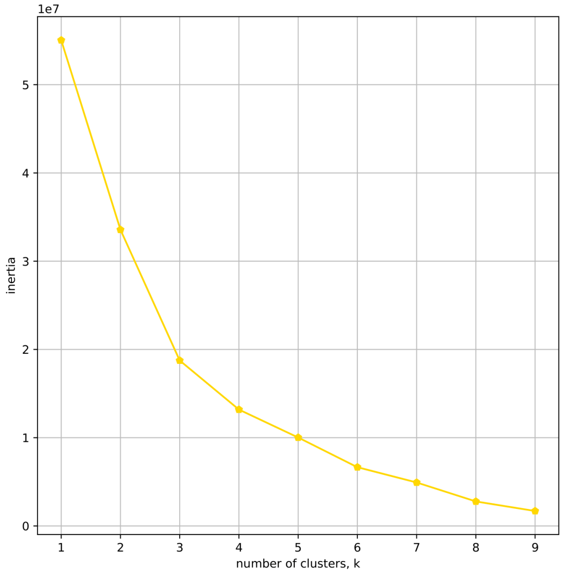

Figure A7: Scree plot suggesting three clusters using the "elbow point" determined by K-Means using 2 PCs (13% of the variance) from k-mer frequencies of three populations, FIN (single origin), CEU (multiple origin), and TSI (single origin). All three populations are of a single superpopulation (EUR).

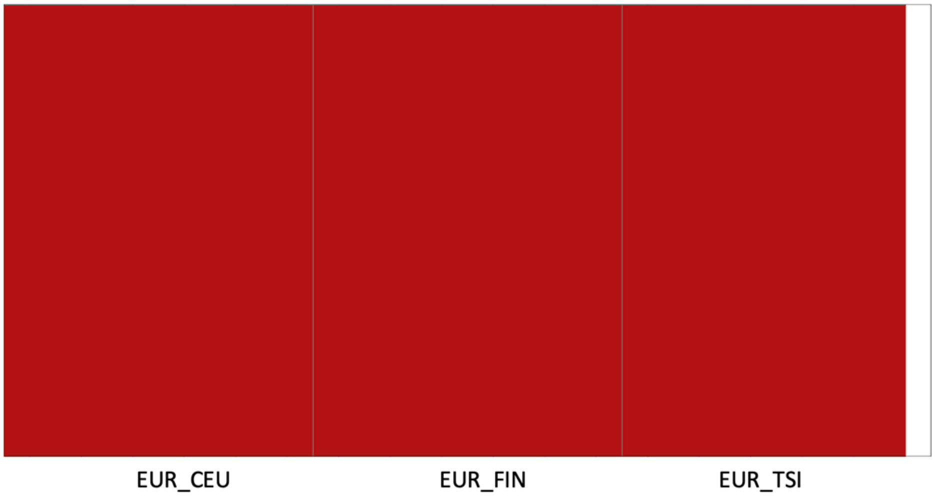

Figure A8: *fastStructure* assignment of individuals into a single population. *chooseK* method determined an optimal K between 1 and 2. fastStructure results for K = 2.

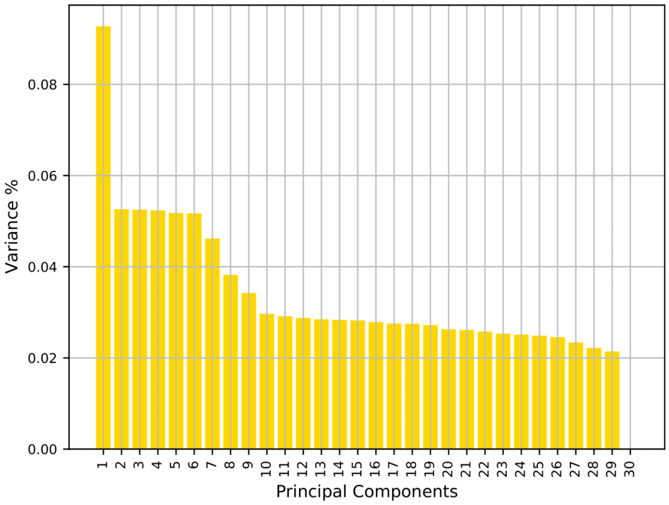

Figure A9: Bar plot showing explained variance per PC in the PCA analysis of 5 superpopulations of single origin.

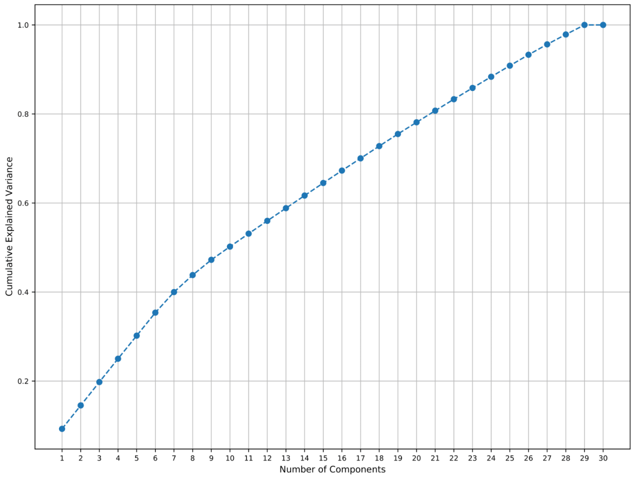

Figure A10: Scree plot showing cumulative explained variance per PC in the PCA in the analysis of 5 superpopulations of single origin.

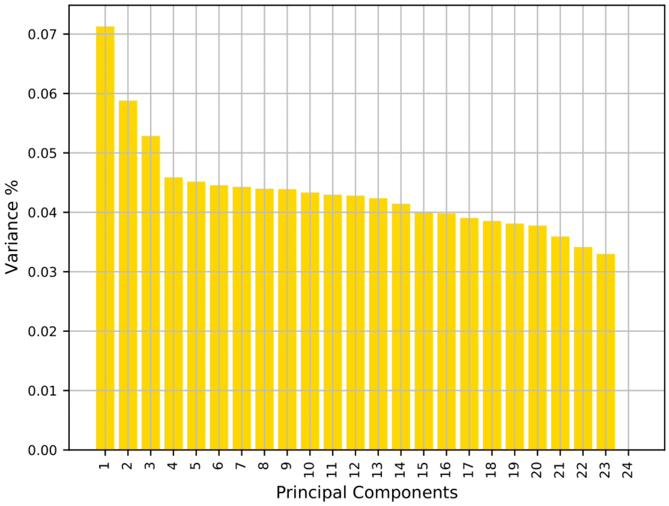

Figure A11: Bar plot showing explained variance per PC in the PCA analysis of 4 superpopulations of single origin (excluding AFR).

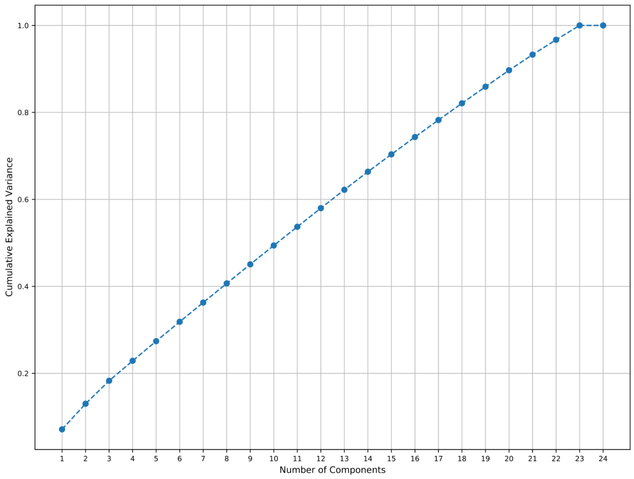

Figure A12: Scree plot showing cumulative explained variance per PC in the PCA analysis of 4 superpopulations of single origin (excluding AFR).

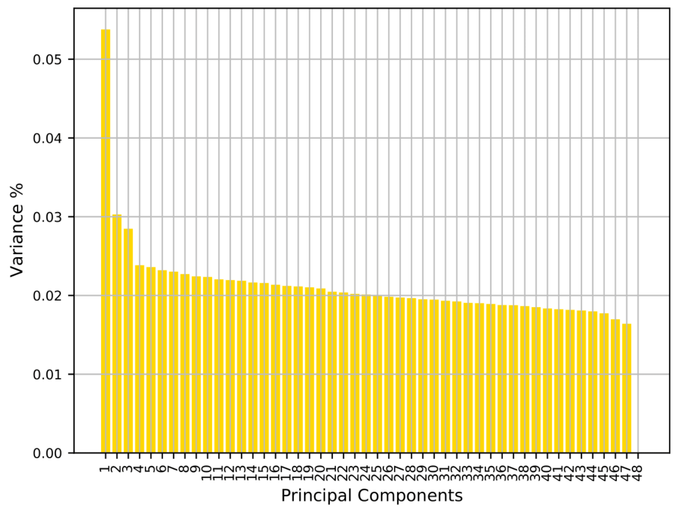

Figure A13: Bar plot showing explained variance per PC in the PCA analysis of 4 superpopulations including samples of single and multiple origin.

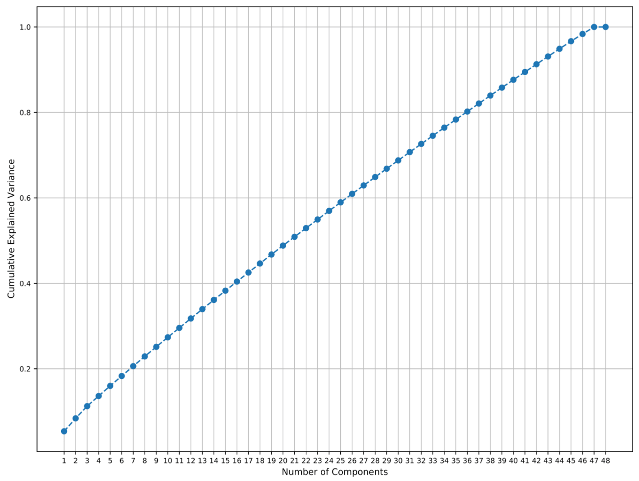

Figure A14: Scree plot showing cumulative explained variance per PC in the analysis PCA of 4 superpopulations including samples of single and multiple origin.

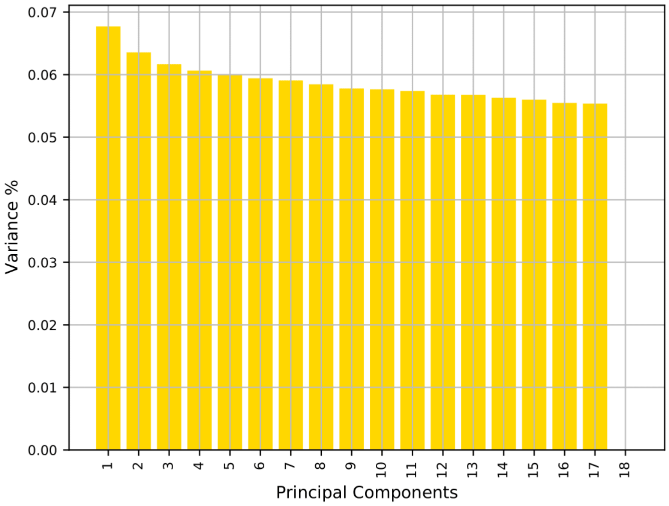

Figure A15: Bar plot showing explained variance per PC in the PCA analysis of 1 EAS superpopulation including samples of single and multiple origin.

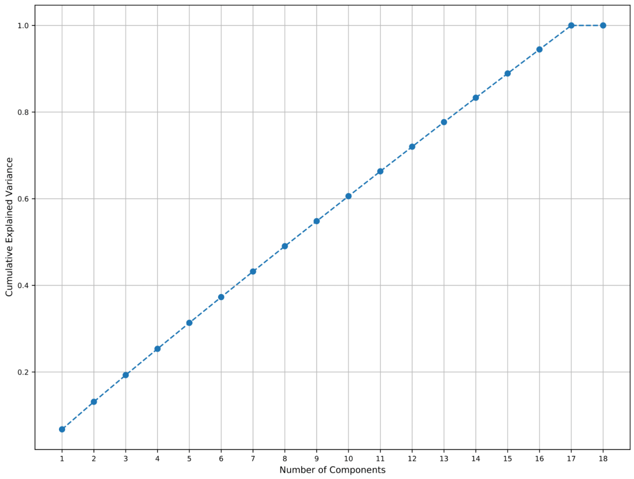

Figure A16: Scree plot showing cumulative explained variance per PC in the PCA analysis of 1 EAS superpopulation including samples of single and multiple origin.

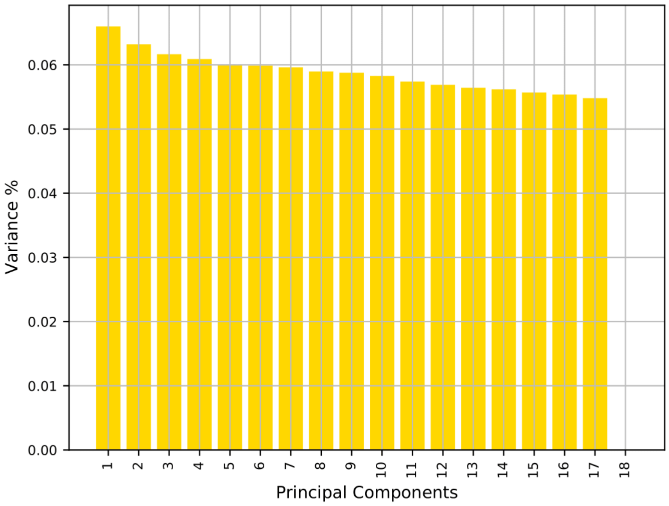

Figure A17: Bar plot showing explained variance per PC in the PCA analysis of 1 EUR superpopulation including samples of single and multiple origin.

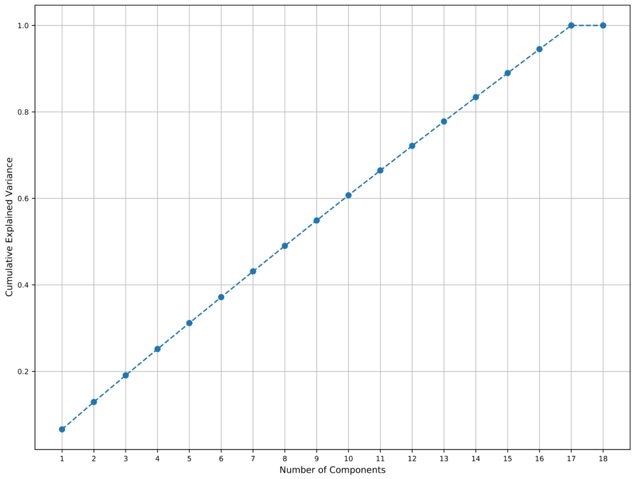

Figure A18: Scree plot showing cumulative explained variance per PC in the PCA analysis of 1 EUR superpopulation including samples of single and multiple origin.

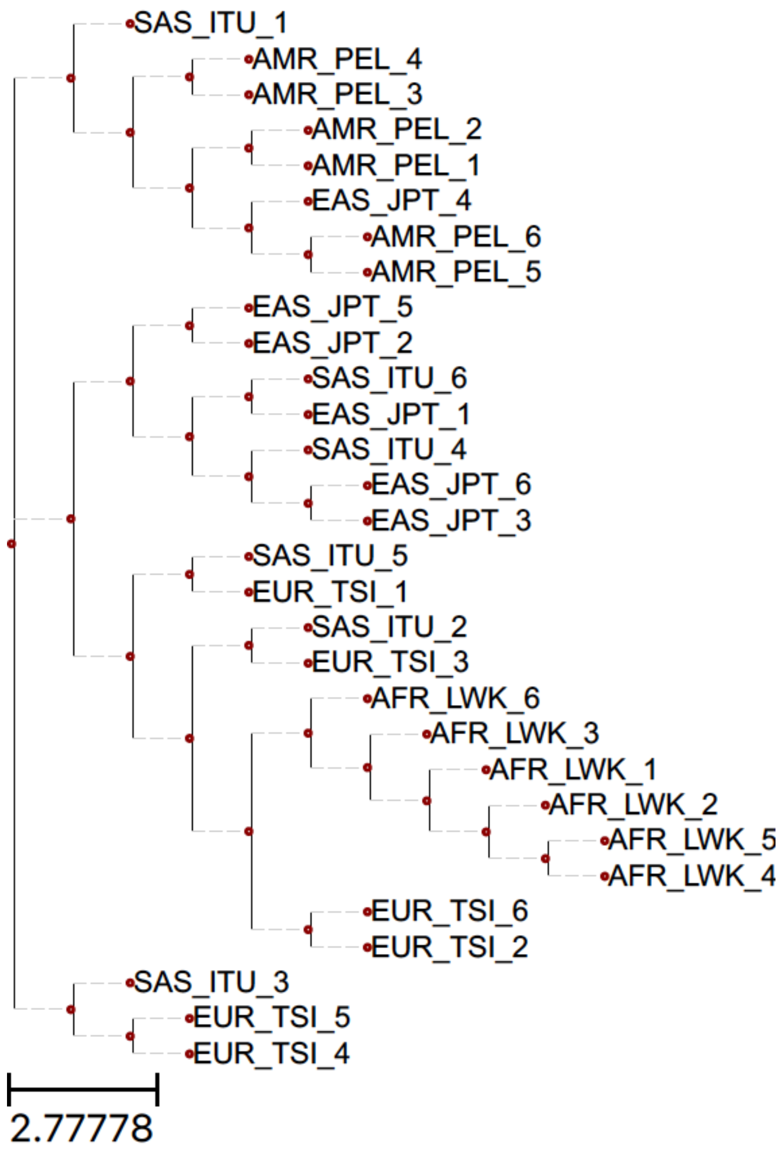

Figure A19: Phylogenetic tree built from pairwise mash distances using k-mer length = 21 and sketch size = 1000 (no monophyly).

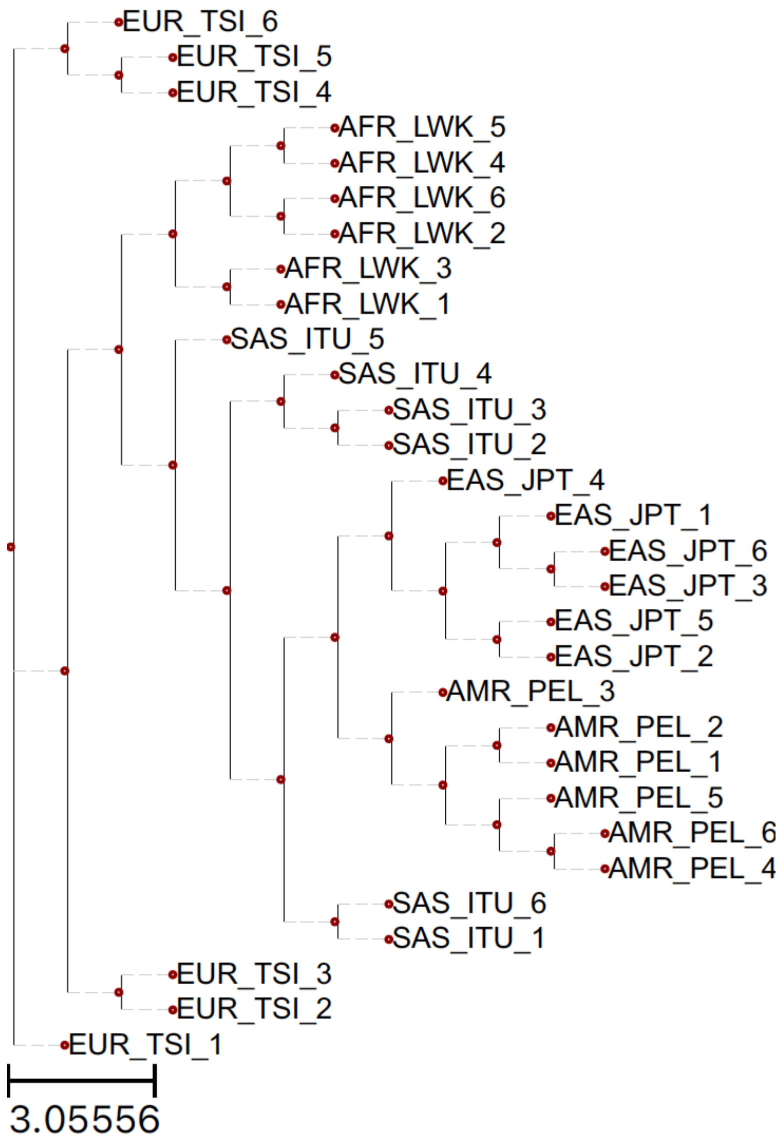

Figure A20: Phylogenetic tree built from pairwise mash distances using k-mer length = 21 and sketch size = 5000 (no monophyly).

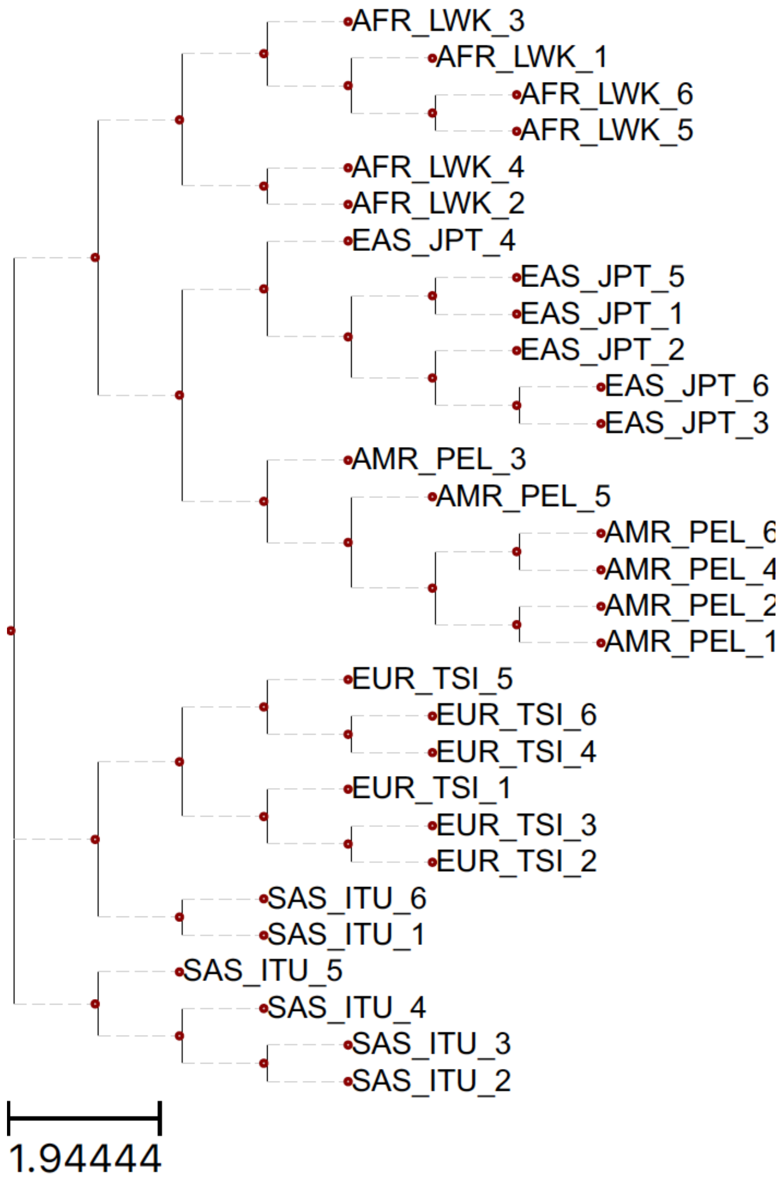

Figure A21: Phylogenetic tree built from pairwise mash distances using k-mer length = 21 and sketch size = 10000 (no monophyly).

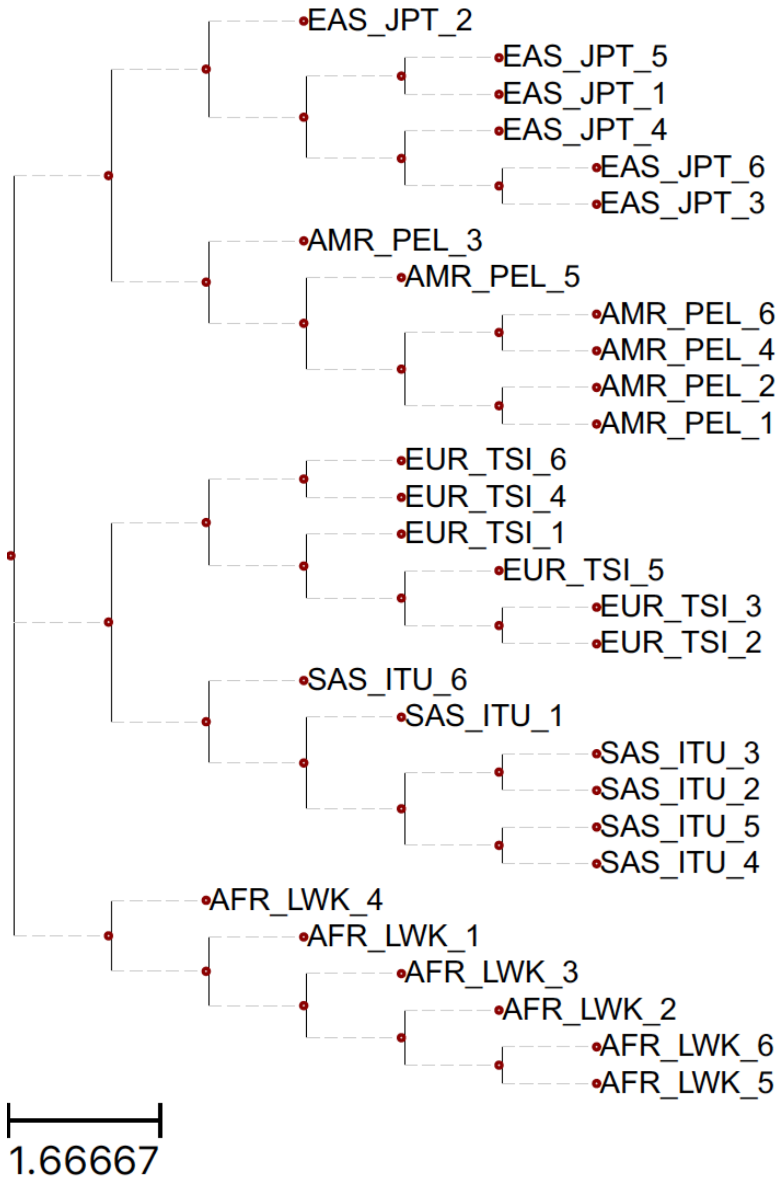

Figure A22: Phylogenetic tree built from pairwise mash distances using k-mer length = 21 and sketch size = 30000 (monophyly observed).
